# Current and future targeted alpha particle therapies for osteosarcoma: Radium-223, actinium-225, and thorium-227

**DOI:** 10.3389/fmed.2022.1030094

**Published:** 2022-11-15

**Authors:** Peter M. Anderson, Vivek Subbiah, Matteo M. Trucco

**Affiliations:** ^1^Department of Pediatric Hematology, Oncology and Bone Marrow Transplant, Cleveland Clinic Children’s Hospital, Pediatric Institute, Cleveland Clinic, Cleveland, OH, United States; ^2^Investigational Cancer Therapeutics, Cancer Medicine, Clinical Center for Targeted Therapy, The University of Texas MD Anderson Cancer Center, Houston, TX, United States; ^3^Division of Pediatrics, The University of Texas MD Anderson Cancer Center, Houston, TX, United States

**Keywords:** osteosarcoma, osteoblastic metastases, bone metastases, lung metastases, radiosensitization, denosumab, IGF1R antibody FPI-1434-225Ac

## Abstract

Osteosarcoma is a high-grade sarcoma characterized by osteoid formation, nearly universal expression of IGF1R and with a subset expressing HER-2. These qualities provide opportunities for the use of the alpha particle-emitting isotopes to provide targeted radiation therapy *via* alpha particles precisely to bone-forming tumors in addition to IFG1R or Her-2 expressing metastases. This review will detail experience using the alpha emitter radium-223 (^223^Ra, tradename Xofigo), that targets bone formation, in osteosarcoma, specifically related to patient selection, use of gemcitabine for radio-sensitization, and using denosumab to increasing the osteoblastic phenotype of these cancers. A case of an inoperable left upper lobe vertebral-paraspinal-mediastinal osteoblastic lesion treated successfully with ^223^Ra combined with gemcitabine is described. Because not all areas of osteosarcoma lesions are osteoblastic, but nearly all osteosarcoma cells overexpress IGF1R, and some subsets expressing Her-2, the anti-IGF1R antibody FPI-1434 linked to actinium-225 (^225^Ac) or the Her-2 antibody linked to thorium-227 (^227^Th) may become other means to provide targeted alpha particle therapy against osteosarcoma (NCT03746431 and NCT04147819).

## Biologic characteristics of osteosarcoma, a bone forming cancer

Pathologic diagnosis of osteosarcoma requires the demonstration of bone formation in the form of osteoid production (1). Despite accurate pathologic diagnosis, genomic instability has resulted in osteosarcomas having heterogeneous molecular signatures, with a relative paucity of actionable molecular targets. Many osteosarcoma tumors and metastases harbor p53 mutations or other mechanisms (e.g., MDM2 amplification) that interfere with apoptosis after damage from standard chemotherapy, newer agents such as tyrosine kinase inhibitors (TKI) of vascular endothelial growth factor (VEGF) (2, 3), and/or radiation therapy (4, 5).

Although osteosarcoma has long been considered relatively radio-resistant (6), this assessment was in the pre-chemotherapy era; radiotherapy has been shown to be more effective against osteosarcoma when given in combination with chemotherapy (5, 7–10) or using proton radiotherapy (11). Another approach that is more biologically effective for bone metastases than conventional low dose fractionated radiation to enhance radiation effectiveness is stereotactic body radiotherapy (SBRT) which delivers precise high dose fractions (12–17). The high Linear Energy Transfer (LET) of alpha particles emitted by ^223^Ra, ^225^Ac, or ^227^Th causes hard to repair double strand breaks, providing another way to potentially overcome the intrinsic biologic resistance of osteosarcoma to radiotherapy (18–20).

## Current therapy of osteosarcoma

The importance of local control measures, especially surgery was shown in a series by Jaffe (21). Current osteosarcoma protocols use variations of the 3-drug (Methotrexate Adriamycin, Platinum, MAP) or 5-drug (MAP + Ifosfamide/etoposide, MAPIE) chemotherapy similar to that reported by the Euramos-1 study (22, 23). The addition of Mifamurtide may also improve outcomes (24–27). Metastatic disease, age > 18 (28) and poor response to neoadjuvant chemotherapy are associated with worse prognosis that to date we have not been able to effectively overcome (29, 30).

Ifosfamide is clearly an active drug in osteosarcoma as shown by its effectiveness against bone metastases and responses in patients not responding to MAP (31). Ifosfamide/mesna can be given with reduced toxicity and improved quality of life when given as an outpatient (32–37). If surgery is not possible or would have an unacceptable effect on the quality of life after response to ifosfamide/mesna, then use of not only radiotherapy with radio-sensitizers (10), but also alpha emitting radiopharmaceuticals such as ^223^Ra can provide options for local and systemic control (12, 17).

## Alpha emitter radium-223 for osteosarcoma

### Osteoblastic phenotype is necessary for bone-seeking radiopharmaceutical targeting against osteosarcoma

An osteoblastic phenotype is often suspected when calcified osteosarcoma metastases are seen on scans. However, active bone formation for the metastases >1 cm should be demonstrated using ^99m^TcMDP bone scan or ^18^FNa bone PET-CT before contemplating use of ^223^Ra in osteosarcoma (12, 17). Better images are obtained when planar images are combined with CT (SPECT-CT). ^18^FNa bone PET-CT has increased sensitivity toward osteoblastic metastases and, because a standard uptake value can be obtained on individual metastases, ^18^FNa bone PET-CT also provides a semi-quantitative assessment of disease burden that can be followed to measure the treatment response (38–46). Radiation is excellent if delivered precisely to tumors avoiding normal tissue. Thus, if there is avid ^99m^Tc-MDP (47) and/or ^18^FNa uptake in osteosarcoma metastases or a local recurrence, then the patient is excellent candidate for the use of ^223^Ra to deliver alpha particle radiation to osteoblastic osteosarcoma tumors and minimal radiation to the surrounding normal tissues, be it adjacent lung, spine, or limb salvage hardware from prior surgeries. If little or no bone formation is seen on these imaging modalities, then the patient is not a good candidate for ^223^Ra.

**FIGURE 1:**
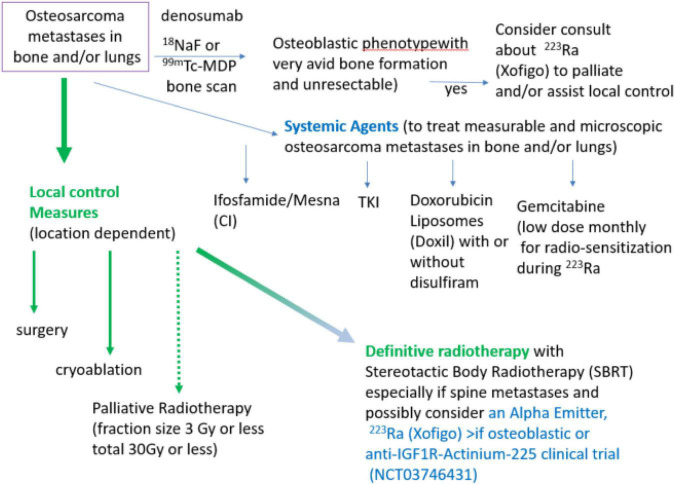
Algorithm of treatment of metastatic, recurrent osteosarcoma with systemic agents, local control measures, and with alpha-emitters ^223^Ra or Anti-IGF1R-Actinium-225. Note if unresectable, referral to a center with expertise in administration of alpha emitters in combination with radiotherapy will be needed.

We have given ^223^Ra in osteosarcoma using the standard dose and monthly infusion schedule of 1.49 microCi/kg intravenously monthly (12) and at 50, 75, and 100 kBq/kg in a dose escalation study (48). From the perspective of the patient, getting ^223^Ra is relatively simple: there is a discussion of the minimal radiation safety requirements (wash hands, flush toilet 2 × because unbound ^223^Ra comes out in the stool), and in our Nuclear Medicine Departments getting ^223^Ra is similar to getting a bone scan injection and takes approximately 10 min. Our current practice is to use the standard ^223^Ra dose on a Wednesday or Thursday to allow gemcitabine to be given as a radio-sensitizer the following day. We also use ^223^Ra in combination with other agents such as denosumab and local control measures in an attempt to both improve the efficacy of ^223^Ra and also to treat areas of metastases that do not have ^223^Ra deposition as illustrated in Figure 1.

### Improving therapeutic index of radium-223 in osteosarcoma

#### Denosumab

Denosumab is a fully humanized anti-RANKL antibody that improves bone density. It is used to treat osteoporosis, reduce skeletal complications of bone metastases, and treat giant cell tumor of bone (49–53). We have made the observation that some osteosarcomas increase the amount of bone formation after denosumab. Thus, monthly denosumab injections during ^223^Ra therapy can increase the amount of ^223^Ra deposited in osteoblastic metastases in osteosarcoma (12).

#### Gemcitabine

Gemcitabine is an excellent radio-sensitizer (10, 54–59). The toxicity of gemcitabine is dependent on not only schedule and dose, but also infusion duration. Shorter infusions (30 min) are associated with less hematologic toxicity than 90 min infusions. Gemcitabine is given daily 5 × had unacceptable mucosal toxicity. Weekly or day 1 and 8 of 3-week cycles are better tolerated. Since gemcitabine must be taken up and phosphorylated to act on the cancer cell, longer infusion times are associated with more hematologic toxicity (60, 61). Giving gemcitabine 600 mg/m^2^ intravenously (iv) once over 30 min 1 day after ^223^Ra is deposited in osteoblastic tumors is a convenient monthly strategy that allows gemcitabine to increase effects within osteoblastic metastases with minimal hematologic toxicity.

##### Case report

**FIGURE 2:**
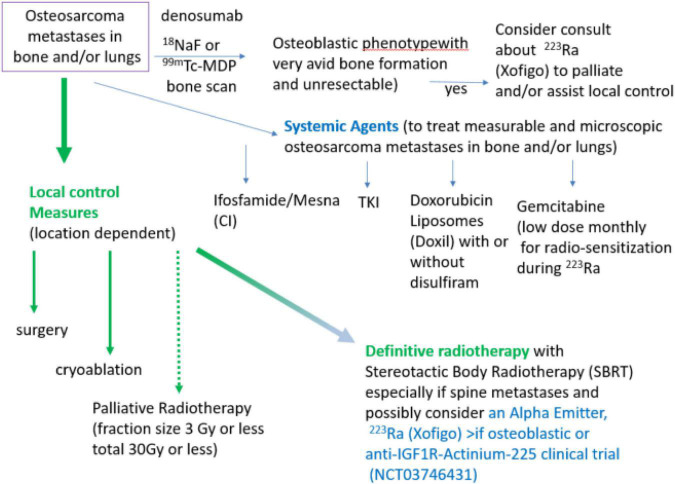
Top shows ^99m^Tc-MDP screening bone scan with avid uptake indicating suitability for alpha particle therapy with the bone-seeking radiopharmaceutical, ^223^RaCl_2_ (Xofigo). ^223^Ra (blue), then gemcitabine (green) next day + monthly denosumab 6 × monthly cycles resulted in improvement, then resolution of symptoms of the unresectable mediastinal and vertebral body osteosarcoma. Currently, the patient is on no therapy and enjoys active lifestyles, such as skateboarding, rock climbing, and skydiving (62).

The following case (Figure 2) illustrates the successful use of ^223^Ra and gemcitabine (62). A 27-year-old patient presented with a large osteosarcoma tumor involving T2-4 extending into both the spinal canal and the left upper lobe. Because of giant cell features, he was initially given denosumab, but when molecular testing revealed FGFR mutation and pathology was reviewed, the diagnosis of osteosarcoma was made. He received 2 cycles of MAP chemotherapy and then because of minimal response was switched to ifosfamide + etoposide. Because the tumor was deemed unresectable, 50.4 Gy over 28 fractions with concurrent ifosfamide + etoposide was given during cycles 5 and 6. He received 2 more cycles of ifosfamide + etoposide then had radiographic progression and clinical worsening (weakness of both lower extremities, some tingling, and need to use a cane). Cardiothoracic, orthopedic, and spine surgeons reviewed his case at the sarcoma conference at the Cleveland Clinic and also deemed the tumor to be unresectable because of the combination of vertebral, spinal canal, and mediastinal involvement. Bone scan with Spect-CT showed avid ^99m^Tc-MDP uptake and he was given 6 monthly cycles of Denosumab, ^223^Ra, followed by gemcitabine. Cytopenias were modest, no transfusions were needed. The patient experienced a clinical response as characterized by increased strength in his legs, no longer requiring a cane to ambulate and resolution of paresthesia. Uptake of ^18^FDG as well and ^99m^Tc-MDP was decreased on repeat imaging. After the response to ^223^Ra monthly 6 ×, he was given oral cyclophosphamide for 6 months (Figure 2). He is now over 9 months off therapy without evidence of recurrence. His activity level has increased and he is able to skateboard (even able to do tricks such as a “treflip,” insert top on Figure 2; Supplementary Video 1), rock climbs often, and has gone skydiving six times.

#### Other chemotherapy agents worth determining suitability in combination with radium-223 in osteosarcoma

Local and systemic therapy is often needed before the logistics of evaluating osteoblastic phenotype and obtaining ^223^Ra for osteosarcoma treatment can be solved. Some active agents in the relapsed metastatic osteosarcoma setting are illustrated in Figure 1 (ifosfamide, TKI, and doxorubicin liposomes). Since many patients have had MAP initially without ifosfamide, ifosfamide with or without etoposide is often the 2*^nd^* line therapy of choice (31–34). When ifosfamide is given with mesna as a continuous infusion, thrombocytopenia, encephalopathy, and renal toxicity are seen less often (33–37, 63). We have also demonstrated that outpatient continuous infusion of ifosfamide + mesna was associated with fewer transfusions and episodes of fever and neutropenia (63). If continuous infusion of ifosfamide + mesna is to be used with ^223^Ra, we would recommend starting 1 day after ^223^Ra administration using a dose of 1 gm/m^2^/d × 1 week. Administration of PEG–GCSF after completion of the ifosfamide infusion is also recommended. This regimen can be repeated every 4 weeks to allow for the combination of the cytotoxic effects when the bone-seeking radiopharmaceutical is most active in bone-forming lesions and to allow for hematologic recovery as ^223^Ra decays.

Tyrosine kinase inhibitors including regorafenib (2, 64) and cabozantinib (3) have efficacy against osteosarcoma. Although a dose adjustment of TKI is sometimes needed to limit skin or GI toxicity, we have found the use of glutamine-disaccharide (Healios) can be helpful in ameliorating GI side effects and helping in eating and nutrition while on these agents (35).

Liposomal doxorubicin (tradenames Doxil or Caelyx) has very low heart toxicity (65, 66). This preparation can be given monthly and has modest hematologic toxicity and is not associated with alopecia when given at 40 mg/m^2^. Thus liposomal doxorubicin has high patient acceptance among relapsed osteosarcoma patients. Cold packs on hands, feet, and the use of glutamine + disaccharide (Healios) can be used to limit hand/foot erythroderma and mucositis/esophagitis, respectively (35). Liposomal doxorubicin is probably most suitable in relapsed osteosarcoma patients who had an initial excellent response to MAP chemotherapy. There is also a clinical trial using liposomal doxorubicin in combination with disulfiram to try to target slowly repopulating cancer stem cells high in aldehyde dehydrogenase (67, 68) (NCT05210374 M. Trucco, PI).

#### Use of local control measures including stereotactic body radiotherapy before or in combination with radium-223

As illustrated in Figure 1, surgery, cryoablation, and/or radiation can provide local control of osteosarcoma metastases. Location and number of metastases (“oligometastatic” is <10) may determine whether to do surgery, cryoablation, or to definitively treat with radiation (e.g., 3 Gy × 20 fractions RT or SBRT 8 Gy × 5 fractions = 40 Gy) or whether palliative radiation (e.g., 3 Gy × 10 fractions) is most appropriate. Reasons to use local control include treatment or prevention of pain as well as reduction of tumor burden, particularly where tumor growth may cause complications (e.g., spine or sacral metastases, and hilar or mediastinal metastases. Unfortunately, for the most common pattern of end-stage metastases (numerous lung metastases) neither whole lung radiation nor ^223^Ra will provide effective doses. Clinical trials such as anti-IGF1R–Actinium 225 (NCT03746431) or Doxil + disulfiram (NCT05210374) would be appropriate in these situations.

### IGF-1R expression in osteosarcoma: An opportunity for anti-IGF-1R antibody-actinium-225 alpha particle therapy

Sarcomas, particularly Ewing sarcoma and osteosarcoma have overexpression of IGF1R (69). Although cold antibody was only modestly effective in Ewing sarcoma and not in osteosarcoma (69), chelation of the alpha emitter ^225^Ac can arm the anti-IGF1R antibody to become a potent alpha emitter (70, 71). Table 1 compares ^223^Ra, which targets areas of bone turnover, with anti-IGF1R-Actinium-225. Currently, the clinical trial NCT0374631 is open at MD Anderson Cancer Center, City of Hope, Memorial Sloan Kettering, University of Minnesota, Dana Farber Cancer Institute, University of Pennsylvania, Juravinski/Hamilton Health, CHU-Montreal, Princess Margaret (Toronto), and CHU Quebec. We expect patients <18 years old to be able to be enrolled when the recommended phase 2 dose is achieved. Thus, the anti-IGF1R-Actinium-225 strategy may be another way to treat osteosarcoma metastases that are not osteoblastic and with alpha-particle radiation that effectively acts at short distances in a powerful manner. Nevertheless, the expression of IGF-1R in normal tissue and/or non-specific binding of antibodies may limit the effectiveness of this approach.

**TABLE 1:** Alpha emitters for osteosarcoma.

Radiopharmaceutical	^223^RaCl2	^225^Ac-anti-IGF1R	^227^Th-Anti Her2
Half-life of radio-metal	11.4 days	10 days	18.7 days
Alpha Particles emitted	4	4	5
Blood clearance	Rapid (<1% at 24 h)	Antibody clearance	Antibody clearance
Radon daughter half-life	4 s	no radon daughter	4 s
Penetration of radioisotope	0.1 mm	0.1 mm	0.1 mm
Imaging with gamma camera	Possible	Not done	Possible
Decays to stable isotope	207-Pb	209-Bi	207-Pb

### Her-2 expression in osteosarcoma: An opportunity for targeted thorium conjugates

Her-2 is expressed in a subset of osteosarcomas. Earlier attempts to target this Her2 expression using trastuzumab were unsuccessful. However, clinical trials using Her2-targeted CAR T-cells suggest that Her2-targeted therapy could be active in osteosarcoma (72). Moreover, better-designed novel antibody drug conjugates like Trastuzumab-Deruxtecan (T-DXd) is showing activity in low Her-2 expressing breast cancers, are also being explored in osteosarcoma. HER2-thorium-227 targeted conjugate (TTC) has recently entered clinical trials in Europe and the USA. “A First in Human Study of BAY2701439 to Look at Safety, How the Body Absorbs, Distributes, and Excretes the Drug, and How Well the Drug Works in Participants With Advanced Cancer Expressing the HER2 Protein” (NCT04147819) is a combination of the alpha-emitting radionuclide thorium-227, an antibody targeting HER2, and a chelator molecule that strongly attaches the thorium-227 to the antibody. This technology harnesses the antibody’s ability to target HER2 by using it to transport the alpha particle emitting thorium-227 to the tumor. Both radium-223 and thorium-227 decay produce alpha particle radiation (Table 1) that causes highly lethal double strand DNA damage in tumor cells, but also useful emission for gamma scintigraphy (73). Although the first in human trial is open for breast and gastric only, the expansion part of the study will include patients with a range of tumor indications with HER2 expression which occurs on osteosarcoma. Only in the context of a clinical trial will it be possible to determine whether benefits for the binding to HER-2 on osteosarcoma outweigh potential toxicity from expression on normal cells and/or non-specific binding of the alpha emitter.

## Summary and conclusion

Alpha emitters have some potent biological advantages that may eventually prove useful for the treatment of osteosarcoma. However, the rarity of this sarcoma and specific situations to test efficacy in randomized clinical trials will be very difficult. Perhaps the use of patients as their own controls with benefit as improved quality of life and/or clinical course better than expected—especially compared to historical controls (74) is possibly the best we can do currently.

## Author contributions

PA: writing of the manuscript and experience with ^223^Ra combination therapy. VS: editing of manuscript and experience with ^223^Ra, anti-IGF1R-Ac225 antibody, and Her-2 TTC. MT: editing of manuscript and current treatment of relapsed osteosarcoma including doxorubicin liposomes with or without disulfiram. All authors contributed to the article and approved the submitted version.

## Supplementary material

Supplementary video 1 Excellent return of lower extremity function after 223-Ra treatment of thoracic paraspinal osteosarcoma as demonstrated by skateboarding.
